# Role of *sapA* and *yfgA* in Susceptibility to Antibody-Mediated Complement-Dependent Killing and Virulence of Salmonella enterica Serovar Typhimurium

**DOI:** 10.1128/IAI.00419-17

**Published:** 2017-08-18

**Authors:** Edna M. Ondari, Jennifer N. Heath, Elizabeth J. Klemm, Gemma Langridge, Lars Barquist, David A. Goulding, Simon Clare, Gordon Dougan, Robert A. Kingsley, Calman A. MacLennan

**Affiliations:** a Swiss Tropical Public Health Institute, Basel, Switzerland; b Novartis Vaccines Institute for Global Health, Siena, Italy; c Institute of Immunology and Immunotherapy, College of Medicine and Dental Sciences, University of Birmingham, Birmingham, United Kingdom; d Wellcome Trust Sanger Institute, Wellcome Trust Genome Campus, Hinxton, Cambridge, United Kingdom; e Institute for Molecular Infection Biology, University of Wuerzburg, Wuerzburg, Germany; f Institute of Food Research, Colney, Norwich, United Kingdom; g Jenner Institute, Nuffield Department of Medicine, University of Oxford, Oxford, United Kingdom; University of California, Davis

**Keywords:** Africa, NTS, Salmonella, antibody function, complement, *sapA*, serum resistance, vaccines, *yfgA*

## Abstract

The ST313 pathovar of Salmonella enterica serovar Typhimurium contributes to a high burden of invasive disease among African infants and HIV-infected adults. It is characterized by genome degradation (loss of coding capacity) and has increased resistance to antibody-dependent complement-mediated killing compared with enterocolitis-causing strains of *S*. Typhimurium. Vaccination is an attractive disease-prevention strategy, and leading candidates focus on the induction of bactericidal antibodies. Antibody-resistant strains arising through further gene deletion could compromise such a strategy. Exposing a saturating transposon insertion mutant library of *S*. Typhimurium to immune serum identified a repertoire of *S*. Typhimurium genes that, when interrupted, result in increased resistance to serum killing. These genes included several involved in bacterial envelope biogenesis, protein translocation, and metabolism. We generated defined mutant derivatives using *S*. Typhimurium SL1344 as the host. Based on their initial levels of enhanced resistance to killing, *yfgA* and *sapA* mutants were selected for further characterization. The *S*. Typhimurium *yfgA* mutant lost the characteristic Salmonella rod-shaped appearance, exhibited increased sensitivity to osmotic and detergent stress, lacked very long lipopolysaccharide, was unable to invade enterocytes, and demonstrated decreased ability to infect mice. In contrast, the *S*. Typhimurium *sapA* mutants had similar sensitivity to osmotic and detergent stress and lipopolysaccharide profile and an increased ability to infect enterocytes compared with the wild type, but it had no increased ability to cause *in vivo* infection. These findings indicate that increased resistance to antibody-dependent complement-mediated killing secondary to genetic deletion is not necessarily accompanied by increased virulence and suggest the presence of different mechanisms of antibody resistance.

## INTRODUCTION

Nontyphoidal salmonellae (NTS) are a major cause of illness and death worldwide (1). While gastroenteritis is the most common clinical manifestation of the disease in the developed world, severe, often fatal disseminated disease is dominant in sub-Saharan Africa, with an estimated global burden of mortality in 2010 of 680,000 (2) and case fatality rates of up to 25% (3–5). The majority of this invasive nontyphoidal Salmonella (iNTS) disease is attributable to serovars Typhimurium and Enteritidis, which account for up to 95% of cases in sub-Saharan Africa (4–6). Several factors contribute to the high prevalence of iNTS disease and associated death in this region, including lack of a definitive clinical presentation, which confounds timely diagnosis (4, 5, 7), coendemic diseases, such as malaria (8, 9) and HIV (10), underdeveloped anti-Salmonella immunity in children (11), and multiple-drug resistance (MDR) (12). MDR may have contributed to the emergence and spread of *S*. Typhimurium genotype ST313, which has been responsible for much of the epidemic iNTS disease in the region (13, 14). The difficulty diagnosing iNTS disease and widespread drug resistance among circulating strains support the need for vaccine development as an effective public health intervention (5, 15).

In regions where iNTS is endemic, acquisition of Salmonella-specific antibodies is important for protection against bacteremia (11, 16, 17). Waning of maternal antibody levels is followed by a peak in iNTS disease at around 1 year of life. Thereafter, a fall in age-related incidence is associated with production of antibody by the child's own immune system (11, 17). These antibodies effect bacterial killing through complement fixation and lysis via the classical and terminal pathways of complement (11) and opsonization of bacteria for uptake by phagocytes and oxidative burst activity (16). Therefore, *S*. Typhimurium virulence and pathogenicity during systemic infection depend in part on the ability to subvert antibody-mediated killing (18). The bacterial surface and its components form an important interface through which they detect and adapt to environmental changes and host immune responses. Surface-associated mechanisms described in *S*. Typhimurium that influence antibody susceptibility include modulation of lipopolysaccharide (LPS) length (19) and the action of proteins that impede complement activity, such as Rck, PgtE, and TraT (20–22). Nevertheless, the mechanistic basis of resistance to antibody-dependent complement-mediated killing (i.e., serum resistance) among Salmonella is poorly understood.

Salmonella enterica serovar Typhimurium ST313 strains exhibit genome degradation similar to that of the human-adapted serovar, *S*. Typhi (13). This process is consistent with the possibility that this genotype is adapting from gastrointestinal toward systemic infection (23, 24), with invasive strains exhibiting decreased enteropathogenicity (25). We and others have reported higher inherent resistance to antibody-mediated killing by complement (26) and macrophages (24) compared to gastrointestinal or non-ST313 strains.

A potential threat to the successful implementation of a vaccination strategy is that further genome modification within the ST313 pathovar could result in adaptation toward enhanced resistance to antibody-mediated killing and subsequently the emergence of potential vaccine escape mutants. Acquisition of such antibody resistance might be accelerated under the selective pressure exerted by an antibody-inducing vaccine. Nevertheless, invasive African *S*. Typhimurium ST313 isolates have mostly remained sensitive to antibody (11, 16), being killed by complement if they remain in the extracellular compartment of blood (27). We hypothesize that increased resistance to antibody and complement-mediated killing in *S*. Typhimurium impacts other functions associated with bacterial survival and virulence, so that the trade-offs between serum resistance and viability help to maintain Salmonella in a serum-sensitive state.

Here, we report the determination of a repertoire of *S*. Typhimurium genes that, when interrupted, result in increased resistance to antibody-dependent complement-mediated killing, using a saturating transposon insertion library screen in conjunction with transposon-directed insertion site sequencing (TraDIS) (28). We subsequently investigated defined *S*. Typhimurium *yfgA* and *S*. Typhimurium *sapA* mutant derivatives in order to gain insight into their biological function and the impact of their absence on infection.

## RESULTS

### Identification of *S*. Typhimurium transposon insertion mutants with enhanced survival in serum.

**FIG 1:**
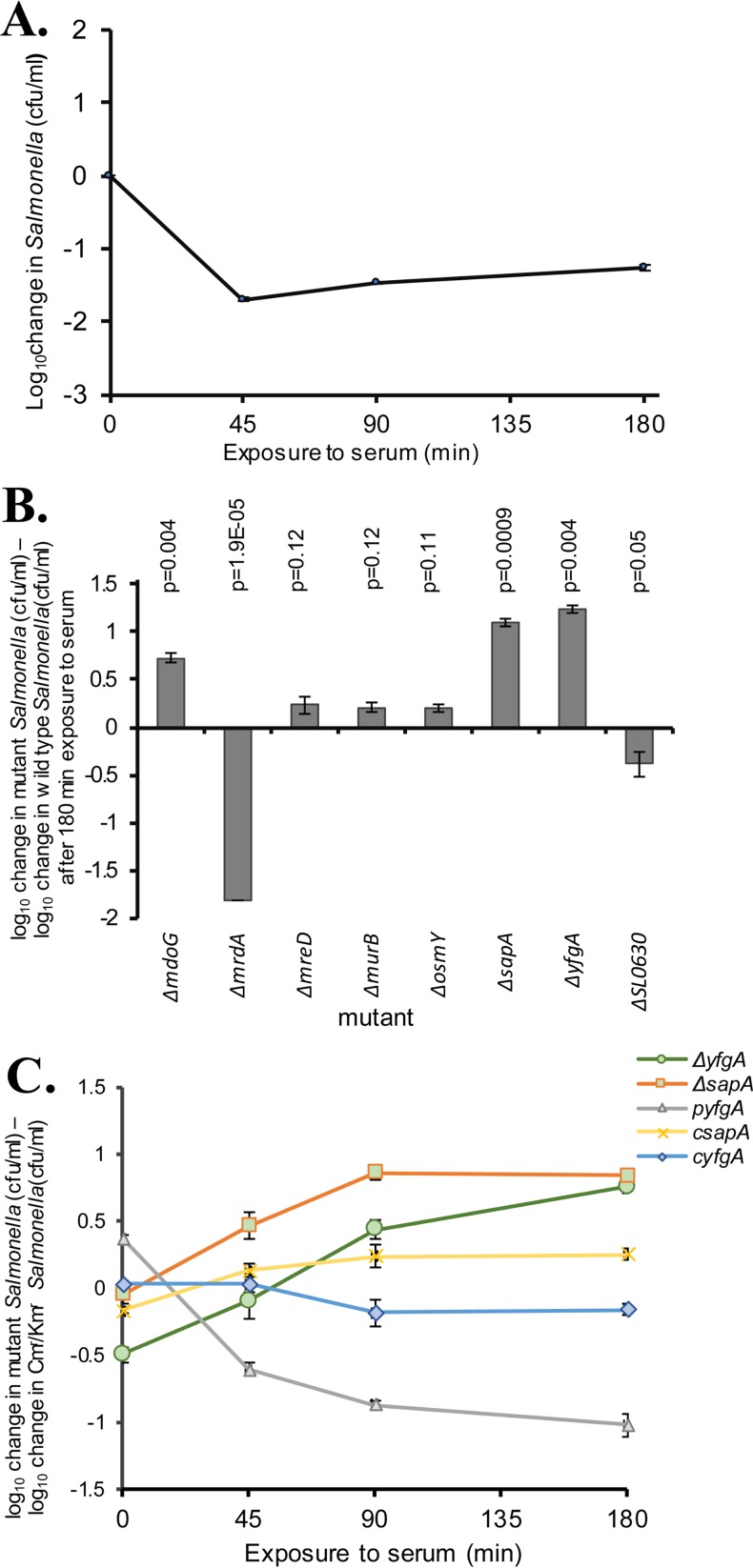
*S*. Typhimurium SL1344 mutants with single-gene deletions exhibiting enhanced resistance to antibody-dependent, complement-mediated killing. All data are from serum bactericidal assays with exposure to immune Malawian adult serum at 37°C for 180 min. (A) Killing of an *S*. Typhimurium SL3261 (SL1344 *aroA* mutant) saturating transposon insertion library with a starting concentration of 10^8^ CFU/ml. Negative values indicate killing. Significantly overrepresented *S*. Typhimurium genes with transposon insertions in the output pool compared with the input pool are shown in Table 1. (B) Resistance to killing of eight mutant *S*. Typhimurium SL1344 strains with definitive single-gene deletions selected from Table 1. Bars represent mean log_10_ changes in levels of mutant Salmonella minus the log_10_ change in wild-type *S*. Typhimurium SL1344 after 180 min of exposure to serum. Positive values indicate resistance to killing. Starting bacterial concentrations were 10^6^ CFU/ml. All means are from three single independent experiments. Error bars indicate standard errors of the means (SEM). (C) Resistance to killing of *S*. Typhimurium SL1344 *sapA* and *yfgA* mutants and corresponding complemented strains in a mixed-inoculum serum bactericidal assay with an *S*. Typhimurium SL1344 strain with intact *yfgA* and *sapA* genes and either a Cm^r^ or Km^r^ antibiotic resistance marker inserted at the *phoN* locus. Strain designations are the following: Δ*sapA*, SL1344Δ*sapA*::*aph*; Δ*yfgA*, SL1344Δ*yfgA*::*aph*; p*yfgA*, SL1344Δ*yfgA*::*aph* pWKS30::*yfgA*; c*sapA* and c*yfgA*, *cis*-complemented strains from Δ*sapA* and Δ*yfgA* mutants (SL1344::*cat*). Strains were mixed at a 1:1 ratio with starting bacterial concentrations of 10^6^ CFU/ml and exposed to immune serum for 180 min. Positive values indicate resistance to killing. All means are from three single independent experiments. Error bars indicate SEM.

To determine genes modulating antibody- and complement-dependent serum killing in *S*. Typhimurium, a saturated transposon insertion mutant library (approximately 9 × 10^5^ independent mutants) was subjected to the bactericidal activity of pooled serum from healthy Malawian adults. Approximately 1 × 10^9^ CFU of the log-phase (input) library was used to ensure that each insertion mutant in the library was represented around 1,000 times in the input mutant pool. After 3 h of incubation with human serum, the viable count decreased to ∼3.5% of the initial viable counts (Fig. 1A). The surviving mutants (output library) were cultured overnight in the absence of serum to permit extraction of genomic DNA from surviving, but not killed, mutants. Genomic DNA was also prepared from the input library Salmonella in order to compare the relative number of insertions at each site in the genome by transposon-directed insertion site sequencing (TraDIS).

A total of 46 genes were significantly overrepresented in the output pool, with a log_2_ read ratio of 2 or more from comparisons of output to input mutant libraries (Table 1). The biological functions of this subset included genes involved in protein/peptide translocation (*sapA*, *sapB*, *sapC*, *sapF*, *tatA*, *tatB*, *tatC*, and *clpX*) and metabolism, including transferases (*ybeA*, *cbiE*, *rfaY*, *ksgA*, *metB*, *mdoH*, *pfkA*, *mreD*, and *metL*) and pentose phosphate pathway enzymes (*deoB*, *zwf*, and *pfkA*). Twenty-three (50%) encoded either membrane protein constituents of the bacterial envelope or proteins involved in synthesis of the bacterial envelope, including peptidoglycan biosynthetic genes (*murF*, *murB*, and penicillin binding protein 2 [PBP2] encoded by *mrdA*) and four of the six known cell-shape-determining genes in Gram-negative bacilli (*mreC*, *mreD*, *mrdA*, and *yfgA*–also known as *rodZ*).

**TABLE 1:** Significantly overrepresented *S*. Typhimurium genes with transposon insertions detected by TraDIS^*b*^

Systematic ID^*a*^	Gene name	No. of reads		Log_2_ read ratio	*P* value	Description
		Input	Output			
SL1344_1088	*mdoH*	1,661	1,825,516	10.018	5.2E−22	Glucans biosynthesis glucosyltransferase H
SL1344_1087	*mdoG*	1,017	1,092,859	9.934	5.30E−22	Glucan biosynthesis protein G
SL1344_4050	*metL*	2,101	255,244	6.858	6.02E−12	Bifunctional aspartokinase II/homoserine dehydrogenase
SL1344_3345	*mreC*	139	14,158	5.899	7.69E−11	Rod shape-determining protein MreC
SL1344_0718	*sucA*	92	10,103	5.732	1.63E−10	2-Oxoglutarate dehydrogenase E1 component
SL1344_3433	*cap*	1,123	54,257	5.474	8.81E−09	Septum formation protein Maf
SL1344_0124	*murF*	10	4,709	5.450	9.98E−16	UDP-*N*-acetylmuramoylalanyl-d-glutamyl-2,6-diaminopimelate–d-alanalanyl ligase
SL1344_0628	*mrdA*	39	5,599	5.358	1.24E−12	Penicillin-binding protein 2
SL1344_1820	*zwf*	219	12,961	5.356	2.86E−08	Glucose 6-phosphate dehydrogenase
SL1344_4496	*deoB*	216	10,604	5.082	5.07E−08	Phosphopentomutase
SL1344_3954	*glnA*	678	23,924	4.949	5.19E−07	Glutamine synthetase
SL1344_3344	*mreD*	83	4,583	4.678	6.92E−09	Rod shape-determining protein
SL1344_1622	*sapA*	100	4,811	4.618	1.79E−07	Peptide transport periplasmic protein
SL1344_2486	*yfgA*	187	6,936	4.616	7.08E−07	Cytoskeleton protein RodZ
SL1344_0630		113	4,968	4.572	8.55E−08	Ribosome-associated protein
SL1344_4489	*osmY*	389	9,390	4.279	1.69E−06	Hyperosmotically inducible periplasmic protein
SL1344_0665	*nagA*	113	3,794	4.192	7.88E−07	*N*-acetylglucosamine–6-phosphate deacetylase
SL1344_0629	*ybeA*	111	2,881	3.820	1.45E−06	(Pseudouridine1915-N3)-methyltransferase
SL1344_4081	*murB*	99	2,347	3.620	1.55E−06	UDP–*N*-acetylenolpyruvoylglucosamine reductase
SL1344_3894	*cyaA*	5,791	67,552	3.522	4.62E−04	Adenylate cyclase
SL1344_3376	*sapG*	270	3,361	3.226	6.53E−04	trk system potassium uptake protein
SL1344_1474		125	1,871	3.131	1.61E−71	Putative multidrug efflux protein
SL1344_3939	*trkH*	1,713	15,780	3.131	6.45E−03	trk system potassium uptake protein TrkH
SL1344_1623	*sapB*	18	909	3.096	6.80E−70	Dipeptide transport system permease protein
SL1344_2209		86	1,198	2.803	5.59E−57	Tail fiber assembly protein
SL1344_1684		385	3,105	2.724	9.59E−54	Putative regulatory protein
SL1344_3929	*tatC*	1,223	8,324	2.671	1.35E−51	sec-independent protein translocase protein
SL1344_4049	*metB*	1,768	10,496	2.504	4.40E−02	Cystathionine gamma-synthase
SL1344_0448		131	1,206	2.499	5.01E−45	Hypothetical protein
SL1344_1442	*dcp*	372	2,505	2.464	9.45E−44	Dipeptidyl carboxypeptidase II
SL1344_0443	*clpX*	509	3,227	2.450	3.23E−43	ATP-dependent clp protease ATP-binding subunit
SL1344_4011	*pfkA*	268	1,910	2.449	3.64E−03	6-Phosphofructokinase
SL1344_2007	*cbiE*	118	1,041	2.388	1.34E−03	Precorrin-6Y C5,15-methyltransferase
SL1344_1693	*narK*	160	1,229	2.354	8.11E−40	Nitrite extrusion protein; MFS transporter
SL1344_3682	*rfaY*	495	2,892	2.330	5.29E−39	Lipopolysaccharide core biosynthesis protein
SL1344_3280		147	1,118	2.302	4.84E−38	Hypothetical protein
SL1344_2377	*nupC*	204	1,303	2.206	7.05E−35	Nucleoside permease NupC
SL1344_3927	*tatA*	408	2,225	2.194	1.73E−34	sec-independent protein translocase protein
SL1344_3726	*slsA*	1,288	6,222	2.187	2.89E−34	Hypothetical protein
SL1344_0091	*ksgA*	187	1,158	2.132	1.63E−32	Dimethyladenosine transferase (adenine1518-N6/adenine1519-N6)-dimethyltransferase
SL1344_3928	*tatB*	1,013	4,552	2.063	2.06E−30	sec-independent protein translocase protein
SL1344_0678	*ybfF*	117	793	2.041	9.67E−30	Putative esterase/lipase
SL1344_1624	*sapC*	47	501	2.032	1.84E−29	Peptide transport system permease
SL1344_4424		4,353	18,008	2.024	3.12E−29	Type II restriction enzyme
SL1344_1236		49	502	2.014	5.87E−29	Putative MutT family protein
SL1344_1626	*sapF*	32	430	2.005	1.08E−28	Peptide transport system ATP-binding protein

a Old locus tags from SL1344 annotation (GenBank accession: NC_016810).

b Significantly overrepresented *S*. Typhimurium genes with transposon insertions were detected by transposon-directed insertion site sequencing (TraDIS) following exposure of an *S*. Typhimurium SL3261 (SL1344 *aroA* mutant) saturating transposon insertion library to immune Malawian adult serum for 180 min. All genes with a log_2_ read ratio of >2 for output reads compared to input reads are included.

To independently verify the impact on antibody-dependent complement-mediated killing of mutation in genes identified in the mutant library screen, we selected eight genes for site-specific deletion in *S*. Typhimurium SL1344 by allelic exchange with an antibiotic resistance gene. Increased representation of particular mutants in the output pool could be due to gene inactivation secondary to transposon insertion, leading to increased resistance to antibody-dependent complement-mediated killing (more likely to be relevant where genes encoded membrane components) or enhanced bacterial growth (more likely to be relevant where genes are primarily involved in metabolism).

First, we selected 20 genes whose transposon insertion mutants were overrepresented following exposure to serum, indicated by the highest ratio of output reads to input reads (>3.5-fold). As the objective of the study was to understand the impact of mutations on resistance to antibody-dependent complement-mediated lysis rather than on bacterial growth, we chose to focus our attention on genes encoding membrane components. For some genes, it proved not to be possible to generate a viable definitive mutant, which could be due to an inherent lack of viability of such mutants. This led to the eight genes selected for generation of definitive mutants. From these eight, *yfgA* and *sapA* mutants were selected for further study based on their enhanced survival in serum compared with that of the other definitive mutants.

We replaced each of eight genes (Δ*mdoG*, Δ*mrdA*, Δ*mreD*, Δ*murB*, Δ*osmY*, Δ*sapA*, Δ*yfgA*, and Δ*SL1344_0630*) with an antibiotic resistance gene by allelic exchange in *S*. Typhimurium SL1344 and tested serum susceptibility of these derivatives individually. Six of the mutants were less sensitive to antibody-mediated, complement-mediated killing than the parent strain, while *mrdA* and *SL1344_0630* definitive null mutants were found to be more susceptible than the wild-type strain, in contrast to the screening data (Fig. 1B). Deletion of two genes in particular, *sapA* and *yfgA*, resulted in a greater than 10-fold decrease in susceptibility to killing by serum.

As these genes had been identified in a screen containing a mixture of mutants, we tested the strains by a second approach using a mixed-inoculum assay with *S*. Typhimurium SL1344 containing a kanamycin or chloramphenicol resistance gene at the *phoN* locus for selection. We determined whether their phenotypes could be complemented by providing the wild-type gene on a plasmid or reconstituting the gene on the bacterial chromosome. The number of viable bacteria with *sapA* or *yfgA* mutations was greater than that of the comparator strain after 1 h of coincubation in serum (*P* = 0.017 and 0.039, respectively) and had approximately 6- to 7-fold more viable counts after 3 h (Fig. 1C). Restoration of the *sapA* gene onto the chromosome resulted in a partial return of antibody sensitivity, and introduction of *yfgA* on a plasmid resulted in an increase in susceptibility to serum relative to the wild type (*P* < 0.01), possibly due to the impact of increased *yfgA* copy number relative to the wild type (Fig. 1C).

### Mutation of *yfgA* and *sapA* likely contributes to decreased susceptibility to antibody-dependent complement-mediated killing by distinct mechanisms.

Loss of functional YfgA and SapA proteins resulted in a similar decrease in susceptibility to antibody- and complement-mediated serum killing. However, the functions of these proteins are distinct. YfgA is a structural protein that plays a role in maintenance of cell shape, while SapA is a component of a peptide transport complex. In order to gain insight into the mechanisms by which the loss of these proteins contributes to antibody susceptibility, we investigated whether increased resistance to serum bactericidal activity was related to increased tolerance to other lytic agents acting nonspecifically on the bacterial membrane.

**FIG 2:**
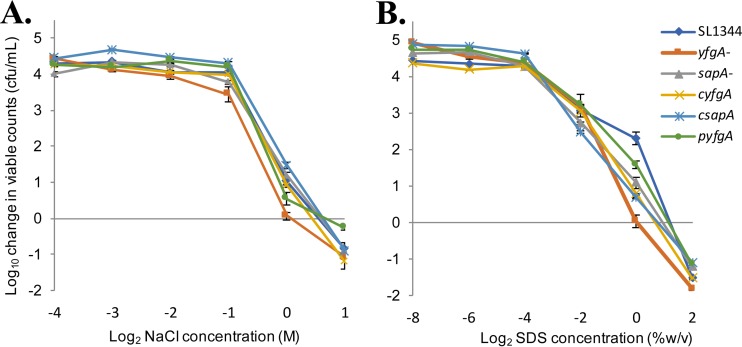
Susceptibility of *S*. Typhimurium SL1344 *yfgA* and *sapA* mutants to osmotic and detergent stress. Shown are log_10_ changes in viable bacterial counts of *S*. Typhimurium SL1344 *yfgA* and *sapA* definitive mutants, complemented strains, and wild-type bacteria following incubation in LB broth containing various concentrations of either NaCl (A) or SDS (B) at 37°C for 8 h. Growth of the *yfgA* mutant, but not the *sapA* mutant, was significantly reduced compared to that of wild-type *S*. Typhimurium (*P* = 0.03 in NaCl and 0.004 in SDS). The concentrations of NaCl and SDS needed to prevent net growth of *S*. Typhimurium in LB broth were significantly lower for the *yfgA* mutant, but not the *sapA* mutant, than for the wild type (1.02 and 1.55 M NaCl, *P* = 0.04; 1.1% and 2.8%, *P* = 0.005). The starting bacterial concentration was 2.5 × 10^3^ CFU/ml. Strain designations are the following: *sapA*^−^, SL1344Δ*sapA*::*aph*; *yfgA*^−^, SL1344Δ*yfgA*::*aph*; p*yfgA*, SL1344Δ*yfgA*::*aph* pWKS30::*yfgA*; c*sapA* and c*yfgA*, *cis*-complemented strains from Δ*sapA* and Δ*yfgA* mutants (SL1344::*cat*). Data represent means from two independent experiments. Error bars indicate SEM.

To this end, we tested the susceptibility of *sapA* and *yfgA* mutants to detergent and osmotic stress. The wild type and mutant derivatives were grown in medium containing dilutions of either NaCl or SDS, and then a change in viable counts was determined after 8 h of incubation. The *yfgA* mutant was more susceptible to both 1 M NaCl (*P* = 0.003) and 1% SDS (*P* = 0.004) than the wild-type equivalent (Fig. 2), while the *sapA* mutant had comparable levels of susceptibility to NaCl and SDS lytic activity to wild-type SL1344, suggesting that the loss of these proteins, leading to decreased susceptibility to lysis by the membrane attack complex, is due to distinct mechanisms.

**FIG 3:**
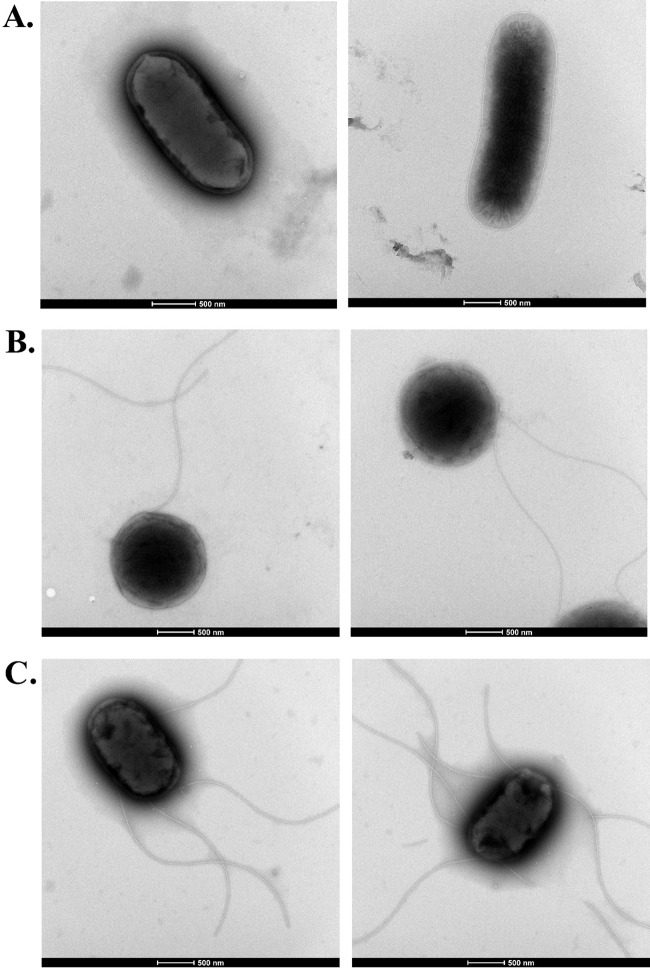
Morphological change in *S*. Typhimurium SL1344 following deletion of *yfgA*. Representative transmission electron micrographs of negative-stained *S*. Typhimurium SL1344 strains. (A) Wild type; (B) *yfgA* mutant demonstrating loss of rod cell shape; (C) complemented *yfgA* mutant, p*yfgA* (SL1344 Δ*yfgA*-pWKS30::*yfgA*). Scale bar, 500 μm.

Further insight into the basis of this difference in mechanism came from the observation that the Δ*yfgA* derivative exhibited profound alterations in cell shape that are likely the result of underlying structural weakness of the cell envelope. Compared to wild-type SL1344, the long axis was markedly absent from the *yfgA* mutant, resulting in spherical bacteria (Fig. 3). Complementation of *yfgA* on a plasmid partially restored cell shape.

### Δ*yfgA* but not Δ*sapA* is associated with alterations in very-long-chain LPS.

**FIG 4:**
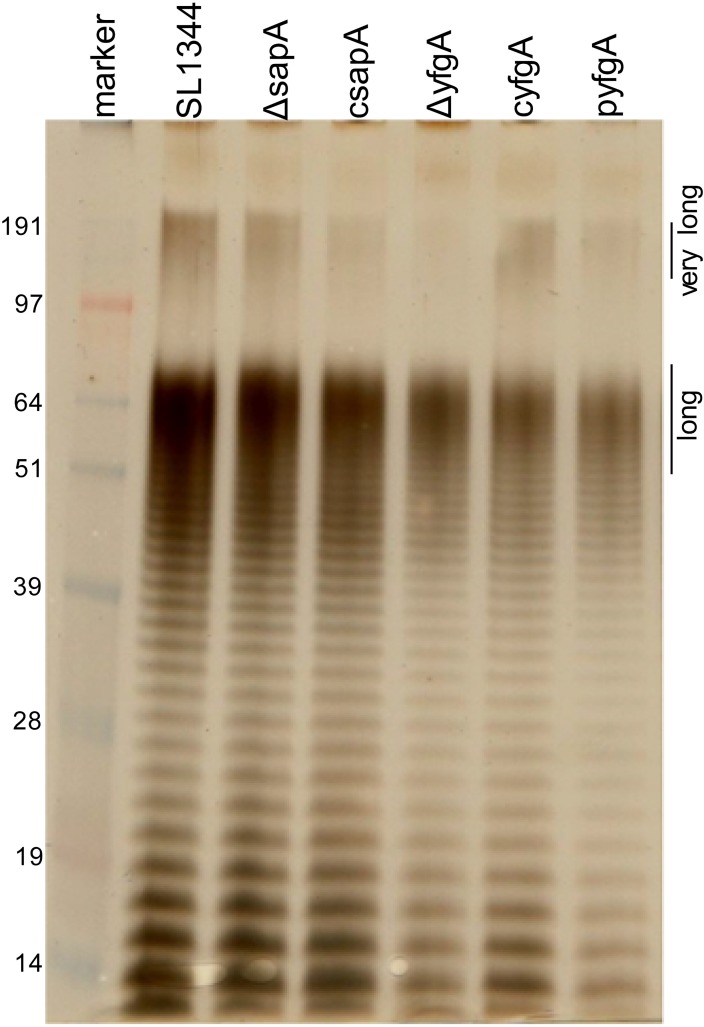
Lipopolysaccharide content of *S*. Typhimurium SL1344 wild type, *sapA* and *yfgA* mutants, and complemented strains. Lipopolysaccharide was extracted from overnight cultures containing equivalent numbers of bacteria from each strain using hot phenol, separated by PAGE on a 12% Bis-Tris gel, and visualized by silver staining. Long- and very-long-chain populations are indicated. Strain designations are the following: Δ*sapA*, SL1344Δ*sapA*::*aph*; Δ*yfgA*, SL1344Δ*yfgA*::*aph*; p*yfgA*, SL1344Δ*yfgA*::*aph* pWKS30::*yfgA*; c*sapA* and c*yfgA*, *cis*-complemented strains from Δ*sapA* and Δ*yfgA* mutants (SL1344::*cat*). Molecular masses of the standard (in kDa) are indicated.

To assess the impact of the absence of the two periplasmic proteins on the gross composition of lipopolysaccharide expression on the outer leaflet of the bacterial envelope, LPS from stationary-phase cultures grown in standard LB broth was extracted from equivalent quantities of each of the derivatives studied and separated by SDS-PAGE. Long-chain LPS was present in all of the derivatives (Fig. 4). However, a high-molecular-weight band corresponding to very long LPS was appreciably less intense in the *yfgA* mutant than in either the wild type or its two complemented counterparts despite similar proportions of short and long O-antigens, suggesting the absence of or a substantial reduction in production of very-long-chain LPS by this strain (Fig. 4). Furthermore, given that LPS was extracted from equivalent quantities of each bacterial strain, it also appears that the *yfgA*-deficient mutant had reduced LPS expression on the cell surface compared to that of the wild type. Deletion of *sapA* did not have an obvious effect on LPS expression.

### Decreased susceptibility to antibody-dependent complement-mediated killing of Δ*sapA* and Δ*yfgA* derivatives is associated with altered interaction with host cells but no increase of *in vivo* virulence.

**FIG 5:**
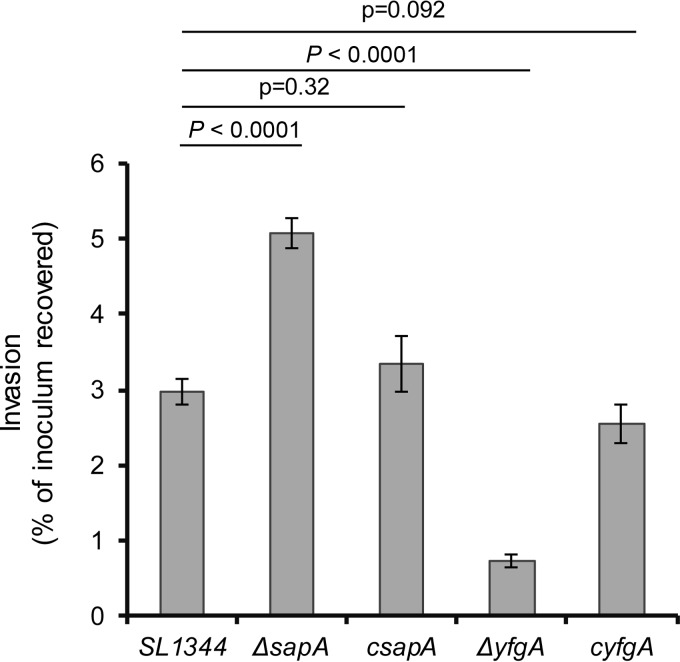
Invasion of enterocytes by *S*. Typhimurium SL1344 wild type, *sapA* and *yfgA* mutants, and complemented strains. Shown are the means and standard errors of viable internalized bacteria as a percentage of the starting inoculum from 6 wells per strain recovered after coincubation with 10^5^/well colon-derived T84 cells for 2 h at an MOI of 10. Two independent experiments were performed in triplicate. *P* values are from *t* test comparisons of means of the 4 derived strains to those for SL1344.

**FIG 6:**
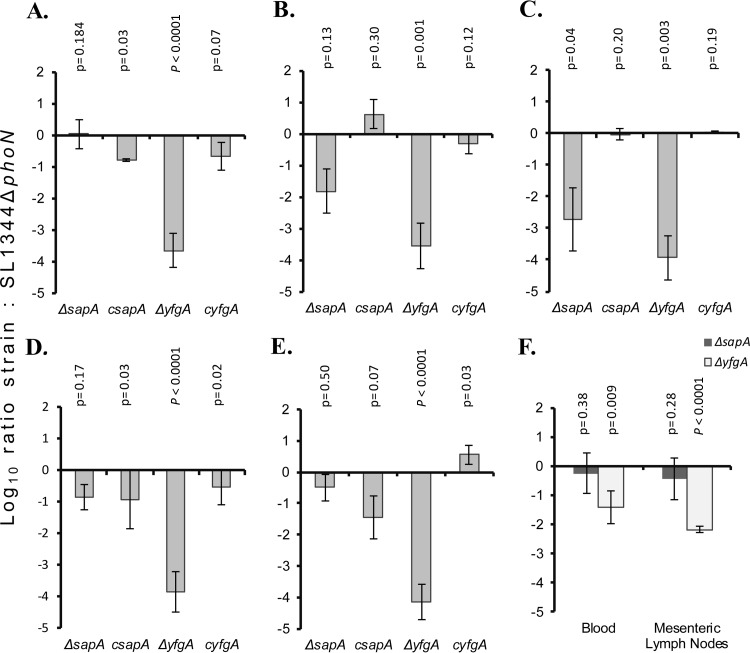
Mixed mouse *in vivo* infections with *S*. Typhimurium SL1344 wild type, *yfgA* and *sapA* mutants, and complemented strains. Mice were infected by the oral route with 5 × 10^8^ CFU of a 1:1 mixture of each mutant or complemented mutant strain and an isogenic *phoN* mutant of *S*. Typhimurium SL1344 carrying an antibiotic resistance marker. Bars represent log_10_ ratios of viable CFU (per gram of organ or ml of blood) recovered from ileum (A), cecum (B), colon (C), liver (D), and spleen (E) after 5 days. (F) Viable bacterial counts from blood and mesenteric lymph nodes were determined from a separate experiment where the complemented strains were not tested. *P* values are from *t* test comparisons of each strain to the SL1344Δ*phoN* strain. Error bars indicate SEM.

Since deletion of *sapA* and *yfgA* in *S*. Typhimurium resulted in enhanced survival in serum, we addressed the question as to how this impacts the interaction of the pathogen with host enterocytes in culture and the ability to colonize the host using a mouse model of Salmonella infection. We first determined the relative ability of Δ*sapA* and Δ*yfgA* derivatives to invade T84 epithelial cells in culture. The Δ*yfgA* mutant derivative invaded T84 cells significantly less (*P* < 0.01) than the SL1344 wild-type parent, while the Δ*sapA* mutant derivative exhibited increased invasion (*P* < 0.01) (Fig. 5). We then determined the ability of Δ*sapA* and Δ*yfgA* mutant derivatives to compete with wild-type SL1344 for colonization of genetically susceptible mice in mixed-inoculum experiments. A 1:1 ratio of mutant to wild type was inoculated into groups of five mice by the gastrointestinal route, and 5 days later the derivatives were enumerated in organ homogenates. The Δ*sapA* mutant derivative was recovered from the colon at more than 10-fold lower levels than SL1344 (*P* = 0.04) but at similar levels from the ileum, liver, spleen, blood, and mesenteric lymph nodes (Fig. 6). In comparison, the *yfgA* mutant was not recovered from any site in the mice 5 days postinfection. Complementation of the gene knockout by replacement of the wild-type copy on the chromosome resulted in nearly complete or complete return to wild-type levels of colonization (Fig. 6).

## DISCUSSION

The current epidemic of iNTS disease across sub-Saharan Africa is significantly associated with the *S*. Typhimurium genotype ST313. *S*. Typhimurium ST313 strains are characterized by genome degradation and pseudogene formation, with similarities to that observed for the human-restricted serovars *S*. Typhi and *S*. Paratyphi A (13), leading to speculation that ST313 is currently in an evolutionary bottleneck which may lead to host adaptation (13). Increasing antibiotic resistance among ST313 strains and the inability to make a clinical diagnosis have hampered antibiotic-based strategies to manage the epidemic of iNTS disease, leaving vaccination as an attractive public health intervention once licensed vaccines against iNTS become available.

Many current vaccine strategies are based on glycoconjugates (15) and are designed to induce protective antibody responses. It is therefore important to understand whether the propensity of *S*. Typhimurium ST313 to deletion and/or loss of function of individual genes will lead to isolates with increased resistance to antibody-dependent complement-mediated killing that could escape vaccine-mediated killing and undermine future vaccine campaigns. To gauge the potential public health threat posed by such mutants, it is also important to ascertain their virulence *in vivo*.

By screening a saturated transposon mutant library, we identified mutants with reduced susceptibility to killing by antibody. Many of these insertions were in genes encoding membrane proteins or proteins involved in the synthesis of cell envelope components or determining cell shape, such as *yfgA*. Other mutations were in genes involved in protein translocation, such as *sapA*, and metabolism. In order to better understand the potential of these mutants to cause disease by escaping vaccine-induced and naturally acquired immunity to Salmonella, we selected *S*. Typhimurium *sapA* and *yfgA* mutant derivatives for further investigation, including assaying their effects on virulence. Despite a similar impact on susceptibility to serum killing, inactivation of these genes had contrasting impacts on virulence and susceptibility to nonspecific cell envelope stress. Deletion of *sapA* resulted in a marked decrease in colonization of the colon of mice but had little impact on colonization of systemic sites. This mutation had little impact on tolerance to osmotic and detergent stress. The *sapA* mutant had an LPS composition similar to that of the wild-type strain but was hyperinvasive for epithelial cells, consistent with previous reports (29). In contrast, virulence was substantially diminished in the *yfgA* mutant, as demonstrated by the inability of the strain to establish infection *in vivo*. This was accompanied by a diminished ability to invade epithelial cells, reduced membrane stress tolerance, and altered LPS composition.

The *sapA* gene is the first 5-cistron *sap* operon and encodes ABC family proteins involved in peptide transport. *sapA* is hypothesized to confer resistance to antimicrobial peptides by shuttling them away from the membrane into the cell, where they are degraded by cytoplasmic proteases (30, 31). Its expression in response to antimicrobial peptides is under the control of the *phoPQ* two-component system (32). A primary role of *yfgA* (*rodZ*) together with other bacterial cytoskeletal proteins, including the actin homolog MreB, is to confer cell morphology in rod-shaped bacteria (33, 34).

The lack of increased virulence *in vivo* or *in vitro* despite increased viability in serum in the *sapA* and *yfgA* mutants corroborates pleiotropic effects from studies of other mutants defective in genes encoding proteins associated with the cell envelope or synthesis of cell envelope components. Deletion of osmoregulated periplasmic glucan (OPG) synthesis genes, *mdoG* and *mdoH*, for instance, results in strains with a mucoid phenotype with resistance to endogenously expressed phage lytic activity, but it renders the strains more susceptible to bile and osmotic stress and diminished virulence *in vivo* (35–37). Insertion mutants in the *mdoG* and *mdoH* genes were also overrepresented in the output library following exposure to serum, and the *mdoG* mutant was significantly less susceptible to killing than wild-type SL1344.

Regarding virulence *in vivo*, *sap* operon mutants of Gram-negative bacteria are known to have diminished capacity for colonization in animal models of infection. In H. ducreyi, for example, *sapBC* mutants are avirulent, while the *sapA* mutant is partially attenuated (38). The *H. influenzae sapA* mutant also has diminished ability to infect rabbits compared to the parent strain (39). In *S*. Typhimurium, attenuated transposon mutants with insertions mapped to the *sap* locus first identified the contribution of these genes to virulence *in vivo* (40). To our knowledge, however, there is no prior description of the specific involvement of the *sapA* gene in *in vivo* survival in a defined null mutant of *S*. Typhimurium. The absence of *sapA* was associated with increased invasion of epithelial cells in culture, a phenotype also described in the H. influenzae *sapA* mutant (29).

Substantial reduction in the ability of the *yfgA* mutant to invade epithelial cells and/or survive osmotic and detergent stress may be an explanation for its inability to colonize mice. Besides altered morphogenesis, absence of other bacterial cytoskeletal proteins is associated with decreased SPI-1 type 3 secretion system protein expression but without loss of the ability to cause systemic infection (41). This may explain the loss of epithelial invasiveness in the *yfgA* mutant. Moreover, the reduction of very long O-antigen in this mutant suggests a role for YfgA in the trimodal expression of LPS O-antigen on the bacterial surface. It is worth noting, therefore, that despite loss of this form of O-antigen, the *yfgA* mutant was still more serum resistant than its wild-type counterpart, especially in view of the defined role of very long O-antigen in conferring resistance to complement (19). While very long O-antigen may be redundant in the presence of long O-antigen chains during complement-mediated killing in *S*. Typhimurium (42), its transient, inducible expression may confer a fitness advantage to both complement killing and elements of nonspecific immunity, such as bile (43, 44). Therefore, while long O-antigen may be sufficient for resistance to antibody and complement in the *yfgA* mutant, the role of very long O-antigen may be required for overall virulence or fitness.

In conclusion, using TraDIS technology, we have demonstrated that deletion of single genes from the genome of *S*. Typhimurium can confer increased resistance to *in vitro* killing by antibody. However, using *sapA*- and *yfgA*-deficient strains as examples, we have shown that their deletion does not necessarily translate to increased virulence *in vivo*. Hence, even as invasive *S*. Typhimurium isolates appear to be adapting to decreased gastrointestinal colonization in favor of systemic infection, emergence and establishment of highly antibody resistant strains may be countered by associated fitness costs.

## MATERIALS AND METHODS

### Bacterial strains and culture.

*S*. Typhimurium strain SL1344 and its derivatives were used throughout this study. SL3261 is a derivative of SL1344 harboring a mutation in the *aroA* gene that has limited impact on susceptibility to serum killing. Bacteria were routinely cultured in Luria-Bertani (LB) broth with either 5 g/liter (low salt) or 10 g/liter (standard) NaCl and were grown aerobically at 37°C. Where appropriate, derived strains were grown in LB media supplemented with either 50 μg/ml of kanamycin, 100 μg/ml of trimethoprim, 30 μg/ml of chloramphenicol, or 100 μg/ml of ampicillin. Isogenic *phoN* mutants *S*. Typhimurium RAK113 (SL1344Δ*phoN*::*cat*) (45) and *S*. Typhimurium RAK072 (SL1344Δ*phoN*::*aph*) were used to distinguish wild-type SL1344 from test strains in competition assays by using antibiotic resistance markers inserted into the *phoN* locus that has no impact on either virulence in mice (46) or serum susceptibility (data not shown).

### Ethical approval.

The study was approved by the College of Medicine Research and Ethics Committee, University of Malawi. Informed written consent was obtained from all blood donors. All animal procedures were performed in accordance with the United Kingdom Home Office Inspectorate under the Animals (Scientific Procedures) Act 1986. The Wellcome Trust Sanger Institute Ethical Review Committee granted ethical approval for these procedures.

### Preparation of serum.

Human blood was drawn from 10 healthy HIV-uninfected Malawian adults and allowed to clot for 2 h at room temperature. Serum was separated from whole blood by centrifugation and stored in aliquots at −80°C until ready for use. For bactericidal assays on defined mutants, serum from a single healthy adult donor, with anti-Salmonella antibody and levels of bactericidal activity similar to those of the pooled serum, was used.

### Transposon insertion library screen and TraDIS.

A mini-Tn*5*-EZ transposon insertion library containing approximately 0.9 × 10^6^ independent insertion mutants in *S*. Typhimurium strain SL3261 (SL1344 *aroA* mutant), described previously (47), was prepared by transferring approximately 50 μl of frozen library to 10 ml LB broth for overnight growth. The culture inoculum contained at least 1 × 10^8^ CFU/ml in order to ensure that individual mutants were not lost from the library and to minimize expansion of the library by culture prior to the experiment. Log-phase cultures were then prepared by inoculating 250 μl of the stationary-phase culture into 25 ml LB broth and grown at 37°C to an optical density at 600 nm (OD_600_) of 0.3 with shaking. The cultures were washed twice in phosphate-buffered saline (PBS) and finally resuspended in PBS to a concentration of 1.7 × 10^8^ CFU/ml. Aliquots of this inoculum were either used directly for genomic DNA preparation (input sample) or exposed to serum killing followed by culture in LB broth before preparation of genomic DNA (output sample). For serum killing, 100 μl of the bacterial suspension was added to 900 μl of neat pooled Malawian serum as described previously and then incubated for 3 h at 37°C with shaking. Viable bacterial counts from serum were then determined by serial dilution and plating on LB agar at 45, 90, and 180 min. The remaining bacteria-serum mixture then was transferred to 25 ml LB broth for expansion of the surviving bacterial mutants overnight at 37°C. Bacteria were resuspended in 9 ml PBS for DNA extraction, and 1 ml of 20% SDS (wt/vol) and proteinase K were added to a final concentration of 200 μg/ml. The suspension was mixed gently and incubated at 37°C for 1 h until a clear lysate formed. DNA was extracted twice using an equal volume of phenol-chloroform-isoamyl alcohol (25:24:1 by volume) each time, followed by final extraction with chloroform. A 0.1 volume of 3 M sodium acetate and 2.5 volumes of ice-cold ethanol were then added to the final aqueous phase to precipitate DNA, which was then washed in 70% ethanol, dried, and resuspended in 500 μl of nuclease-free water.

### Sequence determination and analysis.

Genomic DNA prepared from the transposon insertion library input and output samples were sheared to 300 bp in size, Illumina libraries were prepared, and transposon insertion sites were amplified by PCR as previously described (48). The amplified DNA fragment libraries were sequenced using Illumina GAII sequencer flow cells for single end reads with 54 cycles. The number of sequence reads mapping to transposon insertions was determined by parsing sequence reads containing the terminal transposon sequence. Sequence reads lacking the transposon sequence were not considered further. The transposon sequence was removed from sequence reads, and the remaining sequence was mapped to the reference genome, *S*. Typhimurium SL1344 (FQ312003), using SSAHA. The numbers of reads per insertion site were determined, and a normal distribution was fitted to the log_2_-fold read change normalized for the differences in library read counts for each output sample data set, as previously described (47). The distributions were all shifted to the left (in contrast to the LB passages, where the mean is around 0), suggesting a general reduction in the number of reads observed over the level for most genes. *P* values were then calculated for each gene in each replicate using this as a null distribution. To combine information across replicates, Fisher's method was used, which takes independent *P* values and combines them. This test is known to be anticonservative, so the Bonferroni correction for multiple testing was applied. Genes with a >2-fold increase in sequence reads following exposure to serum and *P* values of <0.05 were considered biologically relevant and analyzed further. Genes with fewer reads observed over the gene in any of the replicates than the average number of reads per gene in the input pool (∼550) were excluded from further analysis.

### Site-directed mutagenesis by allelic exchange.

Mutants were constructed by allelic exchange as described previously (49). PCR fragments for mutagenesis were prepared by amplification of the kanamycin resistance cassette from pKD4 using 70-mer primers (KO_forw and KO_rev; see Table S1 in the supplemental material) consisting of the 50 nucleotides flanking each targeted gene (GenBank accession no. NC_016810) and 20 nucleotides priming pKD4 at the 3′ ends of each primer. Cycle conditions were the following: 95°C for 5 min, followed by 30 cycles of 94°C for 1 min, 60°C for 1 min, and 72°C for 2 min. Approximately 1 μg of PCR product was electroporated into SL1344 harboring plasmid pAJD434, which was grown to log phase at 30°C in LB-trimethoprim broth supplemented with 20 mM l-arabinose to induce recombinase gene expression. Mutants were selected on LB-kanamycin agar. They were then cured of pAJD434 by successive replica streaking on kanamycin and trimethoprim plates and incubation at 42°C to minimize nonspecific recombinase activity. Successful gene replacement was verified by PCR on chromosomal DNA using primers flanking the recombination site, ext_forw and ext_rev (Table S1), and sequencing.

### Serum bactericidal assays on defined mutants.

Log-phase cultures of each strain to be tested were prepared by diluting stationary-phase cultures 1:100 in LB without antibiotics and grown for 135 min. The bacteria were harvested and then washed twice in cold PBS. The pellets were resuspended at an OD_600_ of 0.02 (approximately 10^7^ CFU/ml). Ten microliters of the bacterial suspension was then inoculated into 90 μl of neat, freshly thawed, prewarmed serum and incubated for 3 h at 37°C. Viable bacterial counts were then titrated by serial dilutions and plating on LB agar. Competition serum bactericidal assays were performed in a similar manner with 1:1 mixtures of Δ*phoN* and derived strains and then grown on respective LB-antibiotic plates.

### Complementation experiments.

To complement the *yfgA* mutant in *trans*, its locus plus 274 nucleotides upstream was amplified from SL1344 chromosomal DNA by PCR using the primers *yfgA*_trans_forw and *yfgA*_trans_rev (Table S1). The gene fragment was cloned into pCR2.1 using the TOPO TA cloning kit for subcloning (Invitrogen) and then subcloned into pWKS30. The resulting plasmid, pWKS30::*yfgA*, was transformed into the SL1344Δ*yfgA* strain by electroporation (the resulting strain here is abbreviated as p*yfgA*). The *sapA* mutant could not be complemented in this way despite multiple attempts. Therefore, both mutants were complemented by transduction. To achieve this, a chloramphenicol resistance cassette was inserted into the intergenic regions adjacent to each gene locus in the wild-type strain by allelic exchange as described previously, using the primers *sapA*compl_F, *sapA*compl_R, *yfgA*compl_F, and *yfgA*compl_F (Table S1). Phage P22 was then used to cotransduce the marker and the wild-type copy of *yfgA* and *sapA* genes into the mutant strains. Kanamycin and chloramphenicol replica plates were then used to select Km^s^ Cm^r^ colonies. The presence of the *sapA* and *yfgA* genes in the selected transductants (here referred to as c*sapA* and c*yfgA*, respectively) was further verified by PCR using flanking primers *sapAv*F, *sapAv*R, *yfgAv*F, and *yfgAv*R (Table S1).

### Electron microscopy.

Salmonellae were streaked and grown on LB agar with respective antibiotics. Single colonies were looped directly from an agar plate and resuspended in 100 μl of distilled water to make a slightly turbid suspension. A 10-μl drop was placed on a freshly glow-discharged carbon-coated Formvar grid, and an equal volume of 2% ammonium molybdate with 0.1% trehalose was added for 30 s before draining on filter paper and drying. Grids were imaged on a 120-kV FEI Spirit Biotwin using a Tietz F4.15 charge-coupled device.

### *In vivo* competition experiments.

Groups of 5 female 4-week-old C57BL/6 mice were infected orally with 5 × 10^8^ CFU/mouse of a 1:1 mixture of the mutant strain and an antibiotic resistance-marked strain, *S*. Typhimurium RAK113 (Δ*phoN*::Cm^r^) or *S*. Typhimurium RAK72 (Δ*phoN*::Kan^r^). The interruption of the *phoN* gene has no impact on colonization of the mouse (45, 46), but the alternative antibiotic resistance expressed by these strains and the mutant or mutant complement constructs facilitated enumeration of the strains in homogenized organs following serial dilution and culture on replica agar plates containing each antibiotic in turn. Five days postinfection, a terminal bleed was performed into heparinized tubes, after which the animals were sacrificed and liver, spleen, mesenteric lymph nodes, ileum, cecum, and colon recovered. Organs were homogenized and viable bacterial counts determined by serial dilution and plating on the appropriate antibiotic agar plates.

### Osmotic and detergent susceptibility assays.

Osmotic and detergent susceptibility assays were performed using a modification of a previously described method (50). Briefly, approximately 10^4^ CFU from stationary-phase cultures were inoculated into 4 ml of LB growth medium with increasing concentrations of NaCl or sodium dodecyl sulfate (SDS). Inocula were determined by plating serial dilutions on LB agar. The bacteria were then grown at 37°C for 8 h with shaking at 180 rpm, after which they were titrated by serial dilutions and plating as before. Dose-response curves were obtained by plotting the log_10_-fold change from the initial to final bacterial cell density at each concentration of NaCl or SDS.

### Tissue culture.

To estimate the invasion of T84 cells, epithelial-like cells in culture, we used a gentamicin protection assay. T84 cells were seeded onto 24-well plates at 10^5^/well in Ham's F-12–Dulbecco's modified Eagle's medium (DMEM) mixed 1:1, supplemented with 2 mM l-glutamine and 10% heat-inactivated fetal bovine serum (FBS), and incubated at 37°C for 24 h. Bacteria were grown by inoculating 10 μl of a stationary-phase culture of each strain into 10 ml of low-salt LB broth and incubated statically at 37°C for 18 h. Five hundred microliters of washed bacteria resuspended in the cell culture medium was then inoculated at a multiplicity of infection (MOI) of 10, followed by brief centrifugation and then incubation for 30 min. The inoculum was removed by aspiration, replaced with F-12–DMEM containing 100 μg/ml of gentamicin to kill noninternalized bacteria, and incubated for 90 min. The cells were then washed twice with PBS and lysed with 200 μl of 0.1% Triton X-100 in PBS. Viable internalized bacteria were determined by plating serial dilutions on agar. Invasiveness was assessed by the number of viable internalized or adherent bacteria recovered as a percentage of the inoculum.

### Preparation of LPS and separation by electrophoresis.

Salmonellae were inoculated in 2 ml of LB broth and cultured overnight. The bacteria were then pelleted and resuspended in saline (0.9%, wt/vol, NaCl) to an OD_600_ of 0.5, and 5 ml of this suspension was harvested by centrifugation. LPS was extracted using a modification of the hot phenol method previously described (51). Briefly, pellets were resuspended in 250 μl of SDS-PAGE lysis buffer (2% SDS, 100 mM dithiothreitol, 10% glycerol in 50 mM Tris-HCl, pH 6.8), boiled at 100°C for 15 min, and cooled to room temperature, and then DNase and RNase were added to a concentration of 100 μg/ml. The samples were incubated at 37°C for 1 h, after which proteinase K was added to a final concentration of 200 μg/ml, followed by incubation at 65°C overnight. LPS was then isolated using hot phenol followed by precipitation with 100% ethanol and then resuspended in deionized, autoclaved water. The samples were then separated on a 12% Bis-Tris gel (Invitrogen) and stained using the SilverQuest silver staining kit (Invitrogen) according to the manufacturer's instructions.

### Accession numbers.

Raw sequence data were deposited in the EMBL short read archive under accession numbers ERR305896_1 (input sample), 5850_6 (ERR305895; output replicate 1), 6145_2 (ERR305897; output replicate 2), and 6145_3 (ERR305898; output replicate 3).

## Supplementary Material

Supplemental material
