# Supplementary material for: Role of sapA and yfgA in Susceptibility to Antibody-Mediated Complement-Dependent Killing and Virulence of Salmonella enterica Serovar Typhimurium
Source: Infect Immun. 2017 Aug 18;85(9):e00419-17. doi: 10.1128/IAI.00419-17 (PMC5563563; doi:10.1128/IAI.00419-17)
Supplement: Supplemental material [file supp_85_9_e00419-17__index.html]

Role of sapA and yfgA in Susceptibility to Antibody-Mediated Complement-Dependent Killing and Virulence of Salmonella enterica Serovar Typhimurium — Supplemental material 

# Role of *sapA* and *yfgA* in Susceptibility to Antibody-Mediated Complement-Dependent Killing and Virulence of Salmonella enterica Serovar Typhimurium

## Supplemental material

- Supplemental file 1 -

  Table S1. Primers used in this study.

  PDF, 170K
