# Supplementary material for: Role of sapA and yfgA in Susceptibility to Antibody-Mediated Complement-Dependent Killing and Virulence of Salmonella enterica Serovar Typhimurium
Source: Infect Immun. 2017 Aug 18;85(9):e00419-17. doi: 10.1128/IAI.00419-17 (PMC5563563; doi:10.1128/IAI.00419-17)
Supplement: Supplemental material [file IAI.00419-17_zii999092142s1.pdf]

Table S1. Primers used in this study

Oligo name	Sequence 5’-3’
*murB*_KO_forw	CCAACACAGATACGGTAAACTATTGCCGATTGAGTATCAGGAAAGCAGCCGTGTAGGCTGGAGCTGCTT
*murB*_KO_rev	CCGTCGGCAAGTAGTGAGATCAGCGTCAGGGGAACGGTAGTATCTTTCATCATATGAATATCCTCCTTA
*murB*_ext_forw	TAACCACAAGCCTTCCCAAC
*murB*_ext_rev	GCTCGCCTGAGTGAAACTCT
*osmY*_KO_forw	TTGCCTGAGCTCAAATTACGAGCAAACTATACAGGACAAAATCGATGACTGTGTAGGCTGGAGCTGCTT
*osmY*_KO_rev	GGTGCACATTACGCCTCCCGACAAACGTCGGGAGGACGAATTACGACGAACATATGAATATCCTCCTTA
*osmY*_ext_forw	TGCCTGAGCTCAAATTACGA
*osmY*_ext_rev	GGAGGACGAATTACGACGAA
*mreD*_KO_forw	CATCGCCTTCGGGCGCGACCACGCCGCCTGCGCGTGCGCCGGGAGGGTAAGTGTAGGCTGGAGCTGCTT
mreD_KO_rev	CTGGCGACGCGGGGAACCGGAAGCAAGATACAGAGTTGTCATATCGACCTCATATGAATATCCTCCTTA
*mreD*_ext_forw	GGGATTACTCAGCCATCTGC
*mreD*_ext_rev	GGAACCGGAAGCAAGATACA
*mrdA*_KO_forw	ACCCTTATCACCGTGAGTGATCGTGAAGTCTTGAGAAGATTAAGCAGCGGGTGTAGGCTGGAGCTGCTT
*mrdA*_KO_rev	GATATGAATTTTATCCCAGAAGGTTTTTTTGTTCGGATTATCCGTCATGACATATGAATATCCTCCTTA
*mrdA*_ext_forw	CACCGTGAGTGATCGTGAAG
*mrdA*_ext_rev	CAGTAACGCCAGCAGAATGA
*SL1344_0630*_KO_forw	TTGGCCTTTTTTCTATCGTTATAACGCAATTATTCACCCAGGGGGAAAACGTGTAGGCTGGAGCTGCTT
*SL1344_0630*_KO_rev	ACCCAGTCGGGCATCTTCGTGCCGACAGCGACAAGTTGCAGCTTCACGCACATATGAATATCCTCCTTA
*SL1344_0630*_ext_forw	AACGCAATTATTCACCCAGG
*SL1344_0630*_ext_rev	CGGAAAACGACGCAGATATT
*yfgA*_KO_forw	GGCTCGTCGGCGCCTGAATCCTAATTTACACGTACCTGTAGCTGTAGCGAGTGTAGGCTGGAGCTGCTT
*yfgA*_KO_rev	TTGAATCGGAGCCTGGTTATGCATGAAAAACCTCCCGCGTTACCCGTCTGCATATGAATATCCTCCTTA
*yfgA*_ext_forw	CTAACCTGATGTTTCGCGGT
*yfgA*_ext_rev	GTTGAATCGGAGCCTGGTTA
*sapA*_KO_forw	GGCCGAAGTGCGATACACTTTGCAAATTGAACTTCAAAAACTTAACTATTGTGTAGGCTGGAGCTGCTT
*sapA*_KO_rev	GAAGAAGAGCGTCACCAGCAACAGCAATAACCGACGCAGGGTGAAGATAACATATGAATATCCTCCTTA
*sapA*_ext_forw	GCCAGTTATCCACCGACATT
*sapA*_ext_rev	AGGAAGAAGAGCGTCACCAG
*mdoG*_KO_forw	TAAGCACACAAAGGGGGAAGTGCTTACTTATTATGAAACATAAACGACAAGTGTAGGCTGGAGCTGCTT
*mdoG*_KO_rev	TCACGTTCAGAAAGCAGCAATGCGTCAATATACTCAGTTGTTTTATTCATCATATGAATATCCTCCTTA
*mdoG*_ext_forw	TGGATCGGATCGATATAAGCA
*mdoG*_ext_rev	CTCACGTTCAGAAAGCAGCA
*sapA*compl_F	TTATCTTCACCCTGCGTCGGTTATTGCTGTTGCTGGGTGTAGGCTGGAGCTGCTTC
*sapA*compl_R	AGGCTAAAGCCGATAAAGGTCAGGAAGAAGAGCGTCATGGGAATTAGCCATGGTCC
*yfgA*compl_F	CAAGTACAACGCCAGACATTATTCAACTGACATTGCGTGTAGGCTGGAGCTGCTTC
*yfgA*compl_R	TAGCAGTTGCGCCACGGGGCATTCATTATACCGTGGATGGGAATTAGCCATGGTCC
*sapAv*F	TATCGATCCTTTTCAACGCC
*sapAv*R	GAAATCCAGGCGTCAATGAT
*yfgAv*F	GGGGCATTCATTATACCGTG
*yfgAv*R	GATAAGCTTGAACGCTTCGG
yfgA_trans_forw	GGGGCATTCATTATACCGTG
yfgA_trans_rev	GATAAGCTTGAACGCTTCGG
